# Epidermoid cyst of perineum: a rare case in a young female

**DOI:** 10.1259/bjrcr.20150352

**Published:** 2016-10-07

**Authors:** Uzma Saeed, Naveed Mazhar

**Affiliations:** Department of Radiology, Dallah Hospital, Riyadh, Saudi Arabia

## Abstract

Epidermoid cyst is a common, cutaneous, benign lesion that is frequently seen on the face, neck and trunk. It is rarely seen in the perineal region. The differential diagnosis of perineal cystic lesions includes a wide range of lesions, with an epidermoid cyst being a rare entity to be considered, and only a few such cases have been reported in the literature. Our case was a young Asian female who presented with painless perineal swelling for the past 6 months and was diagnosed as having an epidermoid cyst on the basis of diffusion-weighted MRI and contrast enhancement pattern, which was later confirmed by histopathological findings.

## Background

An epidermal inclusion cyst occurs as a result of implantation of epidermal elements in the dermis. Because most lesions originate from the follicular infundibulum, the more general term of epidermoid cyst is often used. They represent most common cutaneous cysts and are usually asymptomatic; however, they may become secondarily inflamed or infected, or rarely develop malignancy.^1–3^ They occur twice as commonly in males compared to in females^1,2^ and can occur at any age, but are most commonly seen in the third and fourth decades of life.^1^ Epidermoid cysts are typically small, solitary and slow-growing lesions. While they are located commonly on the face, neck and trunk, uncommon and rare sites of occurrence include perineum, extremities, bones and breast.^1,3^

## Case report

A 27-year-old Asian female presented to the surgical outpatient department with complaints of perineal swelling. The swelling was noticed by the patient 6 months ago in the right perineal region; it was approximately the size of a lemon and did not show noticeable increase in size over this time period. It was painless and not associated with changes in the overlying skin colour or texture. The patient was referred for an MRI examination with the clinical query of a possible lipoma in the soft tissues.

**Figure 1.**
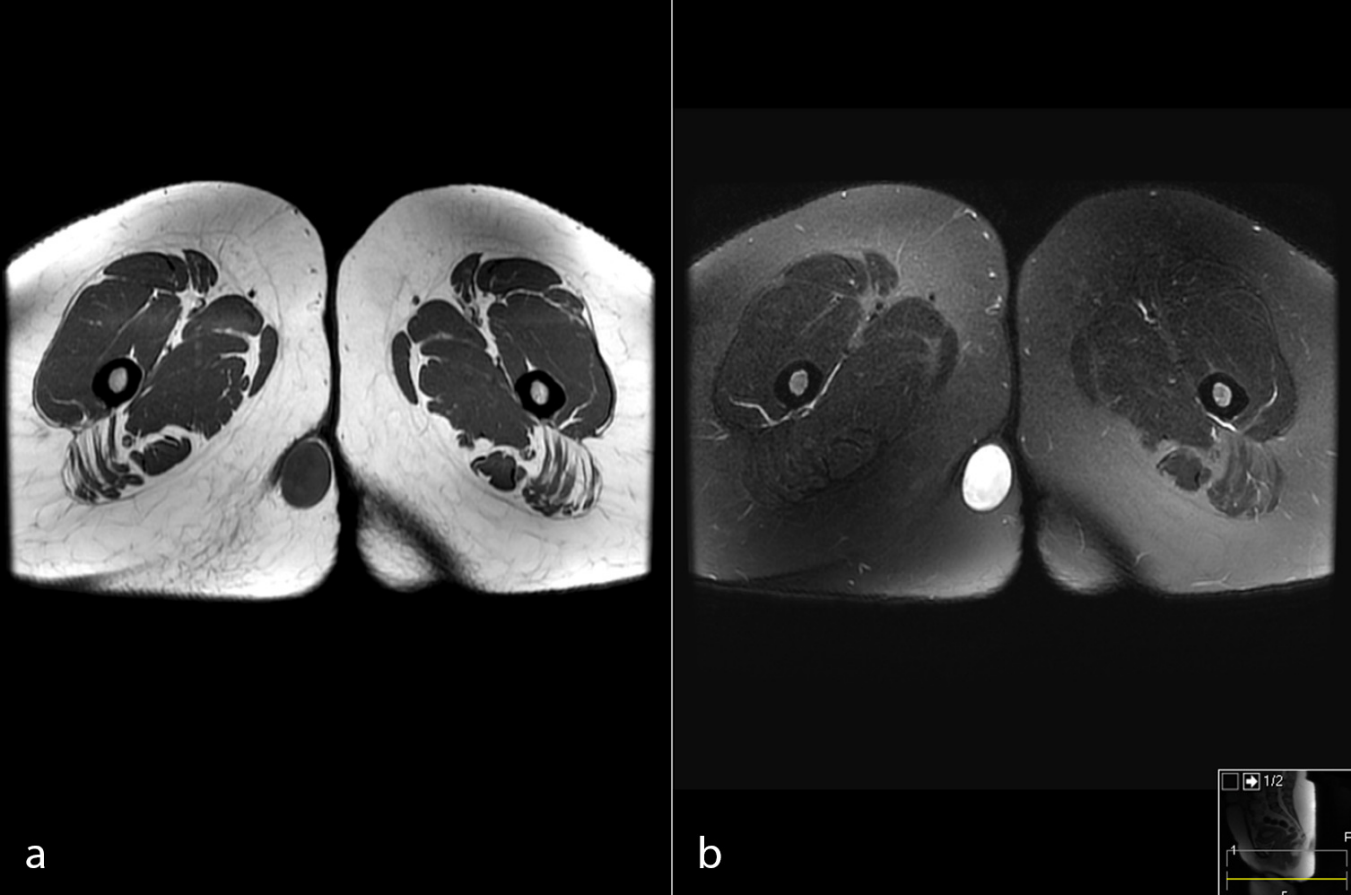
Well-demarcated oval-shaped lesion hypointense to adjacent muscles on axial pre-contrast *T*_1_ weighted image (a) and hyperintense on Proton Density Fat Saturated(PDFS ) image (b).

**Figure 2.**
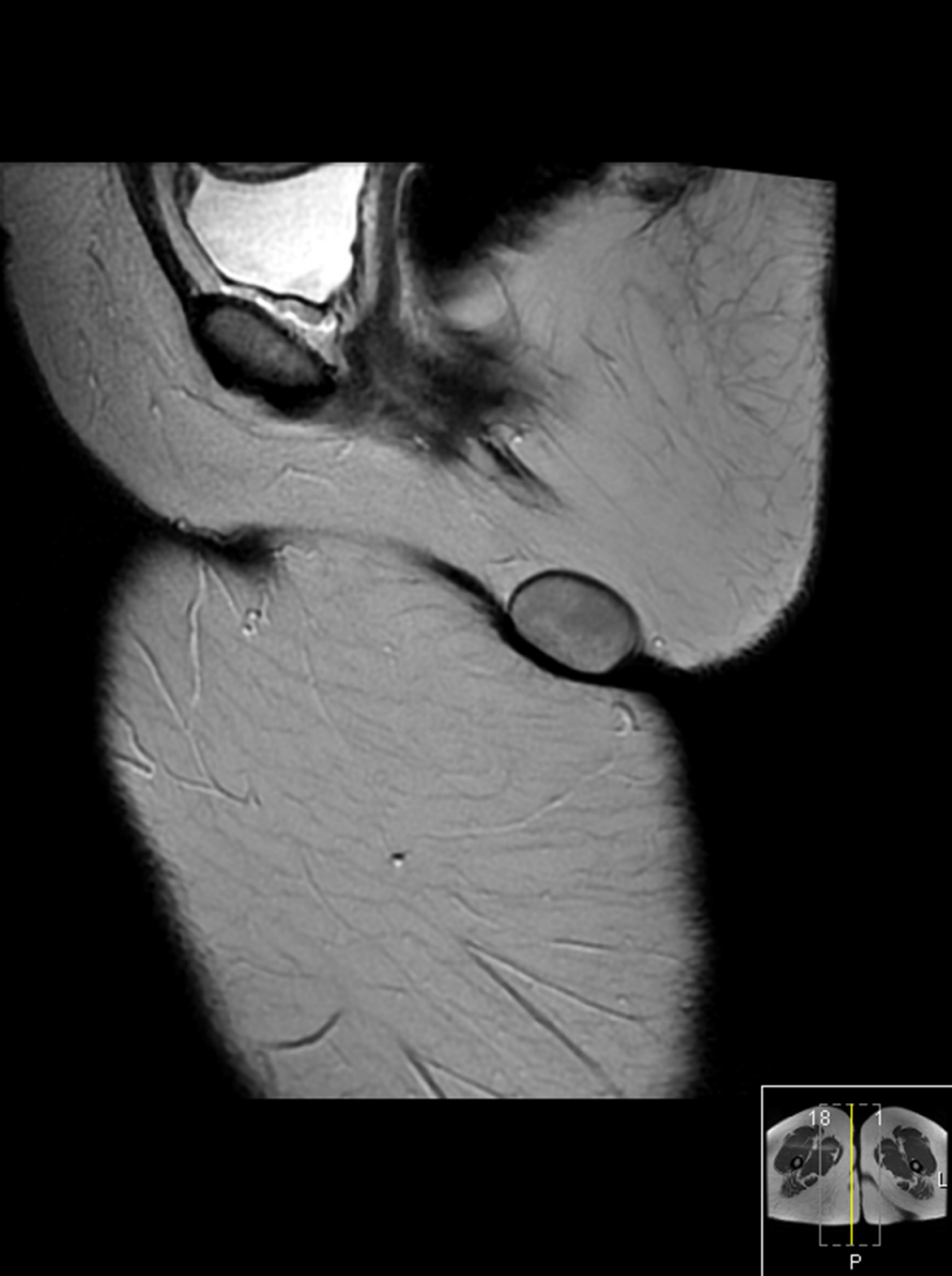
Intermediate signal intensity lesion on sagittal *T*_2_ weighted image.

**Figure 3.**
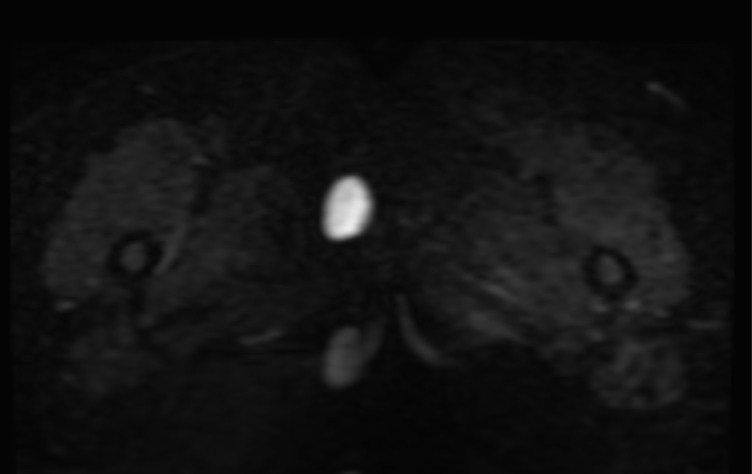
Restricted diffusion elicits diffuse bright signals on diffusion-weighted images (b: 1000 s mm^-2^), confirming the imaging diagnosis of an epidermoid cyst.

**Figure 4.**
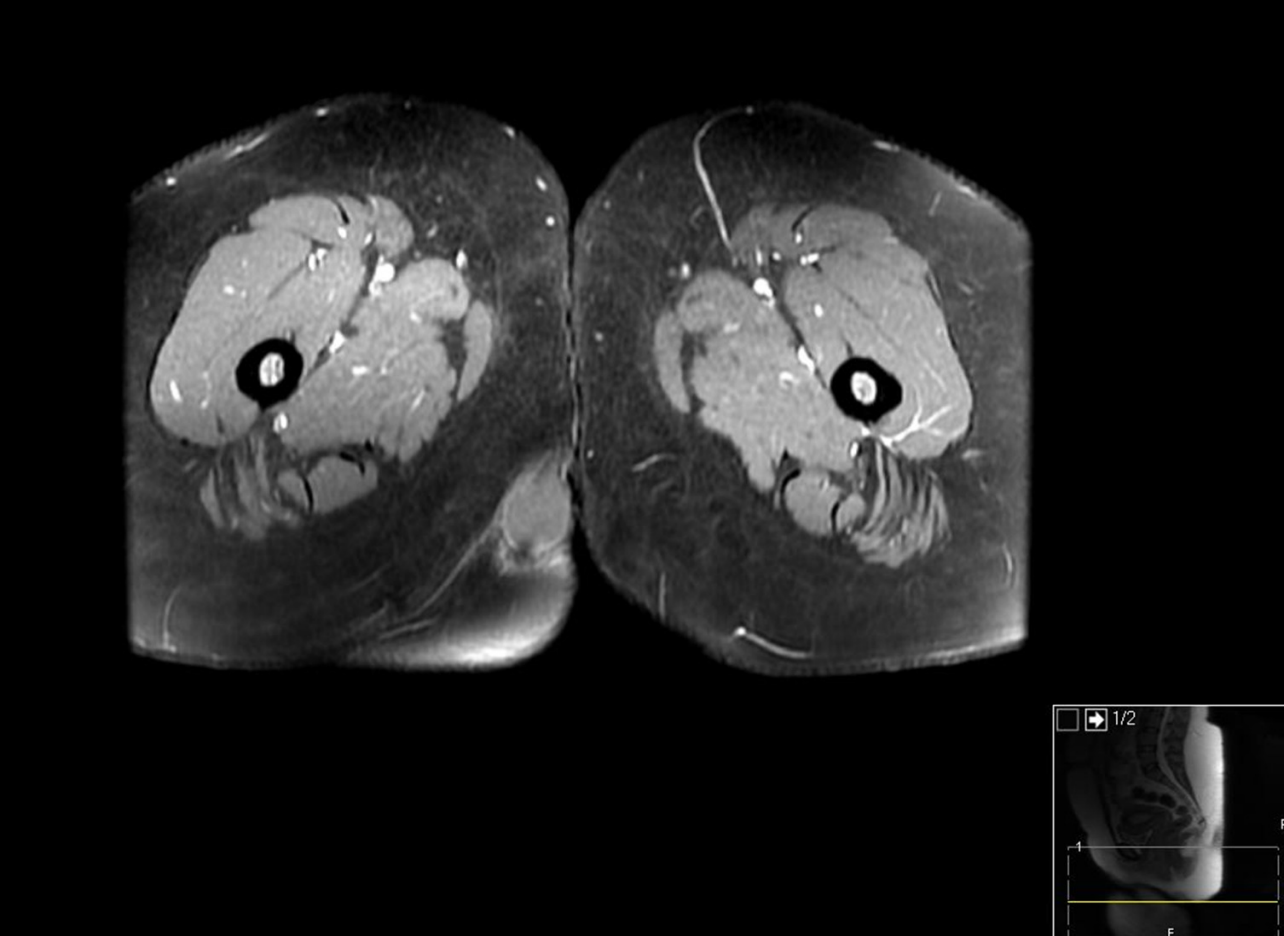
Characteristic thin peripheral post-contrast enhancement of the lesion in the perineum on the right side on axial post-gadolinium *T*_1_ weighted Fat Saturated(FS) image.

MRI of the perineum was carried out before and after administration of intravenous gadolinium contrast. The examination revealed a well-encapsulated lesion measuring 36 × 26 × 24 mm in the subcutaneous soft tissues of the perineum on the right side, appearing as a low signal intensity on *T*_1_ weighted images (Figure 1a), intermediate signal intensity on *T*_2_ weighted images (Figure 2) and heterogeneous high signal intensity on proton density fat-saturated images (Figure 1b). The lesion showed restricted diffusion, appearing as low signal intensity on apparent diffusion coefficient images and as high signal intensity on diffusion-weighted images (Figure 3). Post-contrast images demonstrated subtle enhancement of the lesion wall, with no abnormal internal enhancement (Figure 4). Based on the MRI features, the clinical diagnosis of lipoma was ruled out and epidermoid cyst was suggested as the alternative diagnosis.

1 week later, surgical excision of the swelling was performed and the specimen sent for histopathology, which subsequently confirmed it to be an epidermoid cyst, without any evidence of malignancy.

## Discussion

Epidermoid cysts are slow-growing benign lesions that originate from the epidermal layer of the skin. They occur as a result of proliferation of epidermal cells within the dermis, are lined by stratified squamous epithelium and filled with keratin.^1^ Epidermoid cysts do not usually cause symptoms but can cause discomfort due to their size or if they become infected.

Many factors have been thought to contribute to the formation of epidermoid cysts in general, including ultraviolet light exposure, smoking, human papilloma virus and trauma. In the perineal region, however, mechanical pressure, minor trauma, and even surgical procedures such as episiotomy or needle biopsies may be the main contributing factors.

The differential diagnosis of a cystic lesion in the perineum includes abscess, pilonidal cyst, anal duct/gland cysts, tail gut cyst, benign teratomas and anal/skin cancer.^1^

At present, MRI is the imaging modality of choice to narrow down the differential of soft tissue lesions and can help in establishing a correct diagnosis in cases of epidermoid cysts.^4,5^ Unruptured epidermoid cysts typically display well-defined mass lesions with low signal intensity on *T*_1_ weighted images and high signal intensity on *T*_2_ weighted/fluid-sensitive sequences. Thin peripheral enhancement is characteristically noted after contrast administration.^5,6^ Diffusion-weighted MRI further confirms the diagnosis, as these lesions show significant diffusion restriction.^4,7^

An unruptured epidermoid cyst can show a variable appearance on ultrasound imaging depending upon the size and composition of the lesion. It usually appears as a well-circumscribed, round or oval, and hypoechoic lesion with posterior acoustic enhancement. A larger lesion can be heterogeneous and demonstrates internal bright echoes and filliform anechoic areas. Alternating rings of hypo and hyperechogenicity denoting concentric ring pattern and an echogenic centre with a peripheral hypoechoic rim (target sign) are other ultrasound patterns of epidermoid cysts. Increased blood flow may be seen on Doppler ultrasound at the periphery of the cyst in case of inflammation or rupture.

It may, however, be difficult to differentiate benign from malignant soft tissue masses and histological diagnosis is required in such cases. Histopathologically, epidermoid cysts are lined by stratified squamous epithelium containing a granular layer and filled with keratin.^5,8^

Once the diagnosis of an epidermoid cyst is confirmed, the lesion should be excised with wide free margins. The entire cyst wall should be removed to reduce the risk of recurrence. Prognosis and outcome of these lesions is excellent, with a recurrence rate of only 3%.^5,8^

## Learning points

1. An epidermoid cyst is a common, cutaneous, benign lesion, frequently seen on the face, neck and trunk but rarely in the perineal region.
2. They occur twice as commonly in males compared to females and can occur at any age, but are most commonly seen in the third and fourth decades of life.
3. Differential diagnosis of a cystic lesion in the perineum includes abscess, pilonidal cyst, anal duct/gland cysts, tail gut cyst, benign teratomas and anal/skin cancer.
4. MRI is the imaging modality of choice to narrow the differential of soft tissue lesions and can help in establishing a correct diagnosis.
5. Epidermoid cysts typically are well-defined mass lesions with low signal intensity on *T*_1_ weighted images and high signal intensity on *T*_2_ weighted/fluid-sensitive sequences with characteristic thin peripheral post-contrast enhancement and significant diffusion restriction.
6. Once the diagnosis of an epidermoid cyst is confirmed on histopathology, the lesion should be excised with wide free margins. The entire cyst wall should be removed to reduce the risk of recurrence.

## Consent

Informed consent was obtained from the patient to publish this article, including the images.
